# Supplementary material for: Mechanosensory trichome cells evoke a mechanical stimuli–induced immune response in Arabidopsis thaliana
Source: Nat Commun. 2022 Mar 8;13:1216. doi: 10.1038/s41467-022-28813-8 (PMC8904797; doi:10.1038/s41467-022-28813-8)
Supplement: Supplementary file 1 — Supplementary Information [file 41467_2022_28813_MOESM1_ESM.pdf]

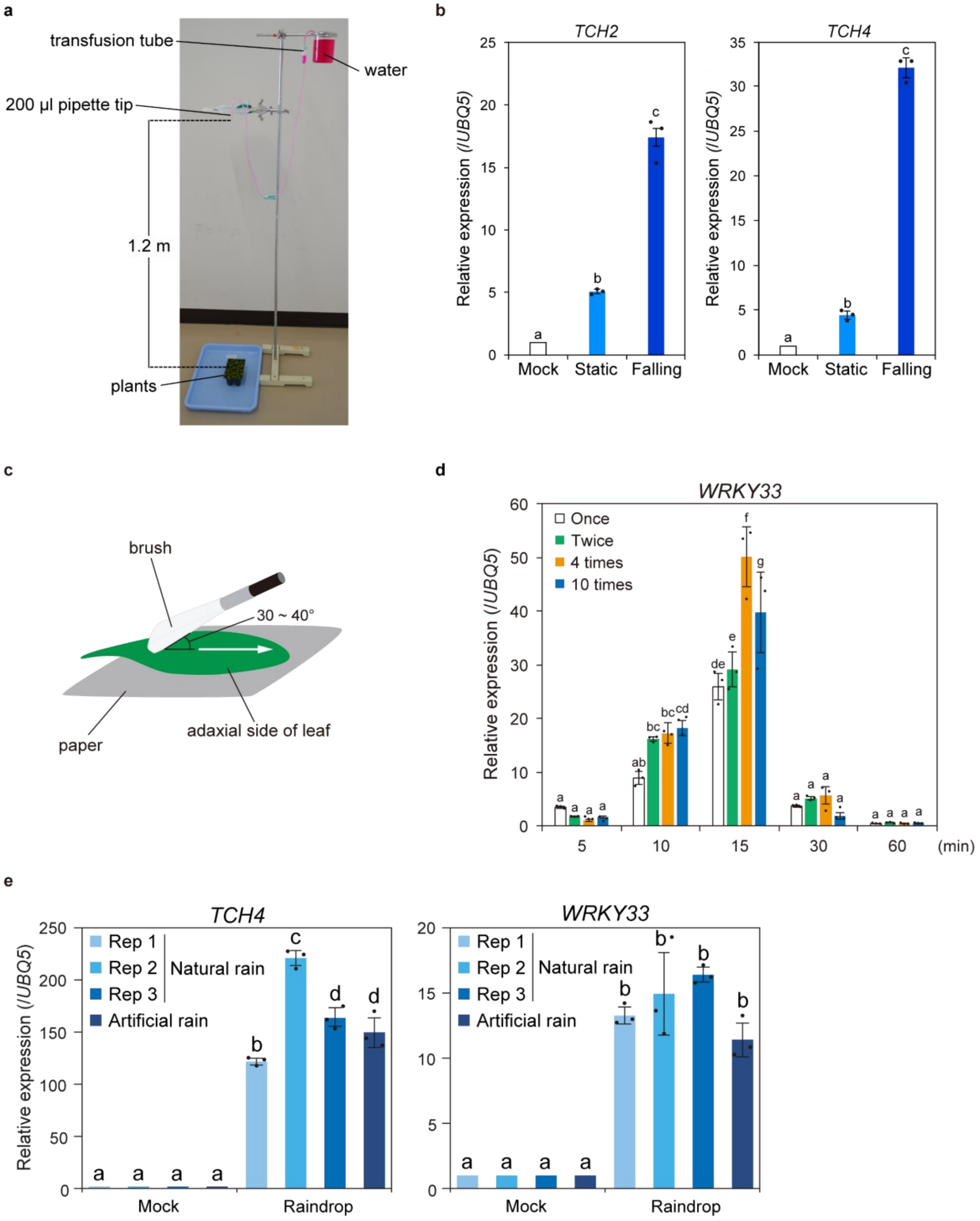
a
transfusion tube
water
200 µl pipette tip
1.2 m
plants
b
TCH2
TCH4
Relative expression (/UBQ5)
Mock
Static
Falling
c
brush
30 ~ 40°
paper
adaxial side of leaf
d
WRKY33
Once
Twice
4 times
10 times
Relative expression (/UBQ5)
(min)
e
TCH4
WRKY33
Rep 1
Rep 2
Rep 3
Natural rain
Artificial rain
Relative expression (/UBQ5)
Mock
Raindrop

Supplementary Fig. 1 | Raindrops and MS initiate the transient expression of defence-related genes. a The device drops 13 μL water droplets from a height of 1.2 m onto the adaxial side of leaves. **b** The adaxial side of wild-type leaves was treated with water droplets and collected after 15 min. The expression levels of *TCH2* and *TCH4* were significantly higher in response to 1 falling raindrop (falling) than to 1 water droplet directly placed on the leaf surface (static). n = 6 plants examined over 3 independent experiments. Each dot indicates a technical replicate. Data are presented as mean ± SD. Different letters above bars indicate significant differences (one-sided Tukey's multiple comparison test; $P < 0.05$). **c** Brushing method. The adaxial side of leaves from 4-week-old plants was brushed along the main vein at an angle of 30-40°. **d** The adaxial side of wild-type leaves was brushed for the indicated number of times. The maximum level of *WRKY33* expression was detected 15 min after brushing. Mean ± SD. Different letters above bars indicate significant differences (one-sided Tukey's multiple comparison test; $P < 0.05$). n = 6 plants examined over 3 independent experiments. Each dot indicates a technical replicate. **e** Four-week-old Col-0 plants were treated with falling natural rain for 3 min (3 biological replicates), or 10 falling artificial raindrops. The rainfall at that time was 1 (mm/hr). Transcript levels of *TCH4*, and *WRKY33* 15 min after treatment were determined using RT-qPCR and normalized to *UBQ5*. Data are presented as mean ± SD. Different letters above bars indicate significant differences (one-sided Tukey's multiple comparison test; $P < 0.05$). n = 6 plants examined over 3 independent experiments. Each dot indicates a technical replicate.

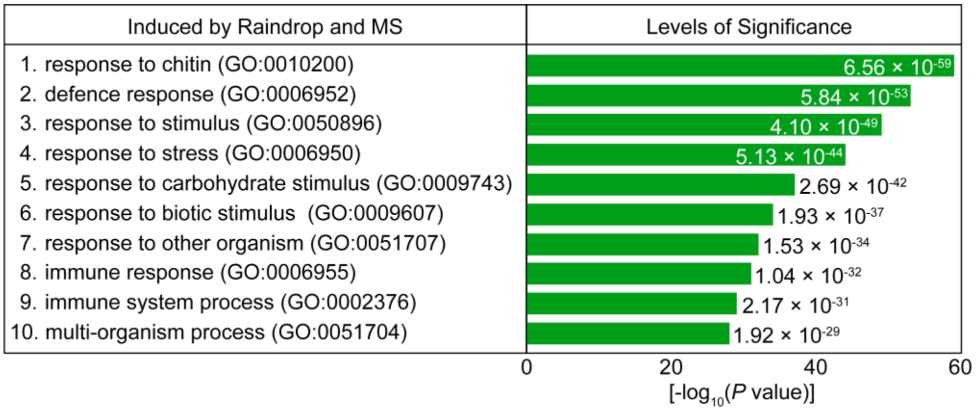

Supplementary Fig. 2 | Raindrop- and MS-induced 917 genes was enriched in GO categories associated with stress responses. Gene Ontology (GO) categories enriched in 917 genes induced by brushing. Enrichment of GO categories for the biological process was determined using BiNGO. The table shows GO terms with *P* values (one-sided hypergeometric test) from the lowest to the tenth.

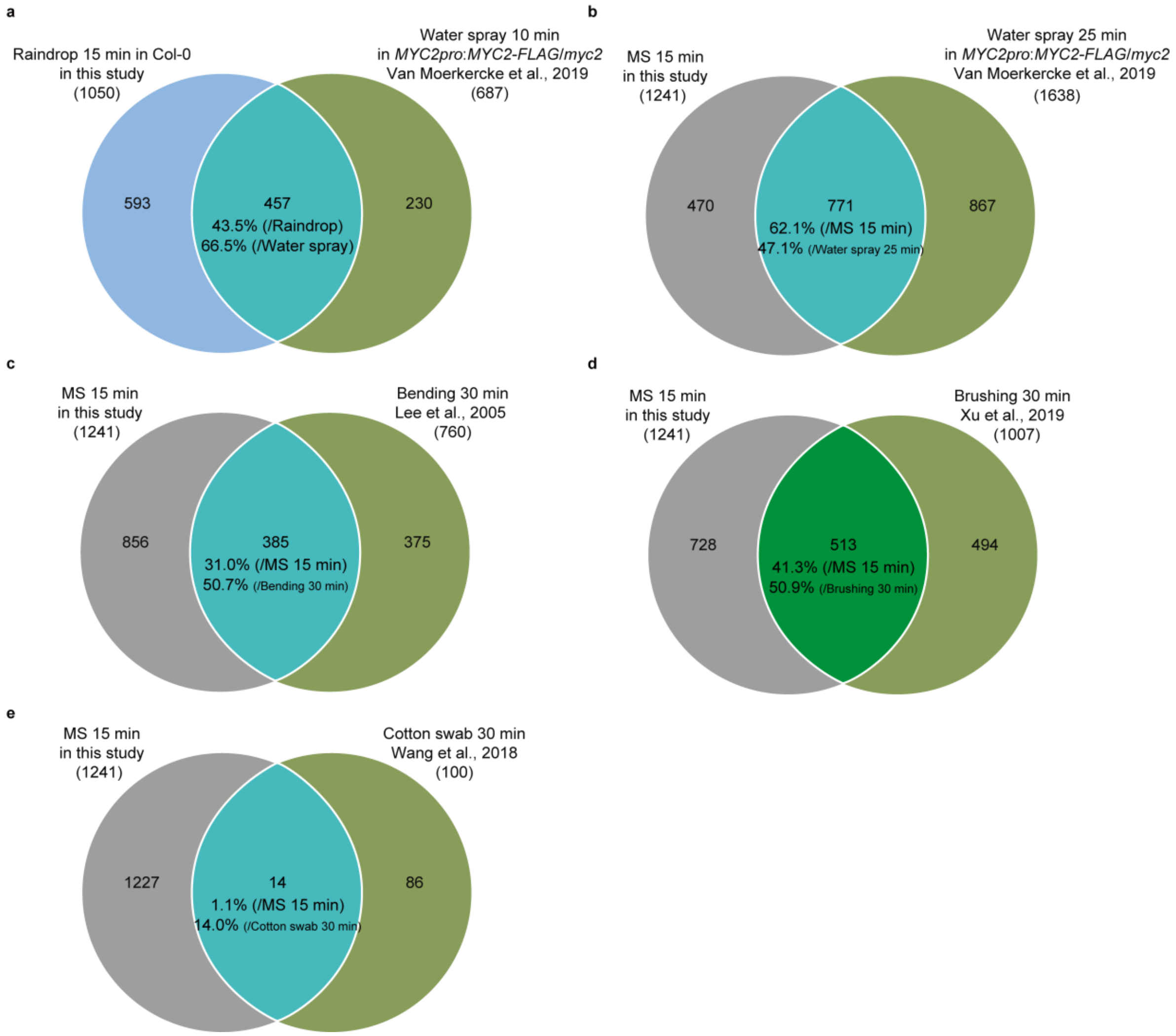

Supplementary Fig. 3 | Comparison of transcriptome datasets of this study with those shown in previous reports. a Venn diagram of the overlap between transcriptome datasets from 1,050 genes upregulated 15 min after raindrop treatment in Col-0 [$\log_2$ fold changes ($\log_2$FC) ≥ 1, $P < 0.05$] and 687 genes upregulated 10 min after water spray in *MYC2pro*:*MYC2-FLAG*/*myc2* ($\log_2$FC ≥ 1, $P < 0.05$). Pearson correlation coefficient (r = 0.556281244). **b-e** Venn diagrams of the overlaps among transcriptome datasets from 1,241 genes upregulated in 15 min after MS ($\log_2$FC ≥ 1, $P < 0.05$), 1,638 genes upregulated in 25 min after water spray ($\log_2$FC ≥ 1, $P < 0.05$), Pearson correlation coefficient (r = 0.556021735) (b), 760 genes upregulated in 30 min after bending ($\log_2$FC ≥ 1, $P < 0.05$), Pearson correlation coefficient (r = N.D.) (c), 1,007 genes upregulated in 30 min after bending ($\log_2$FC ≥ 1, $P < 0.05$), Pearson correlation coefficient (r = 0.263571236). (d), and

100 genes upregulated in 30 min after cotton swabbing ($log_2FC \geq 1$, probability ≥ 0.08), Pearson correlation coefficient ($r = 0.606250705$) (e). These figures were obtained from transcriptome profiles in this study and previous reports (Supplementary Data 11) using Venny (https://bioinfogp.cnb.csic.es/tools/venny/).

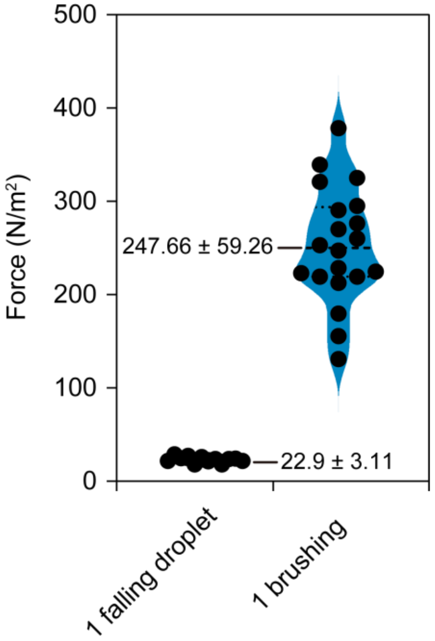

Supplementary Fig. 4 | Force measurement applied to the leaf surface by falling droplet or brushing. Falling 1 droplet and 1 brushing (MS) apply the force 22.9 ± 3.11 (N/m^2) and 247.66 ± 59.26 (N/m^2), respectively. 1 falling droplet: n = 12, 1 brushing: n = 20 trials examined over 3 independent experiments. Each dot indicates a technical replicate.

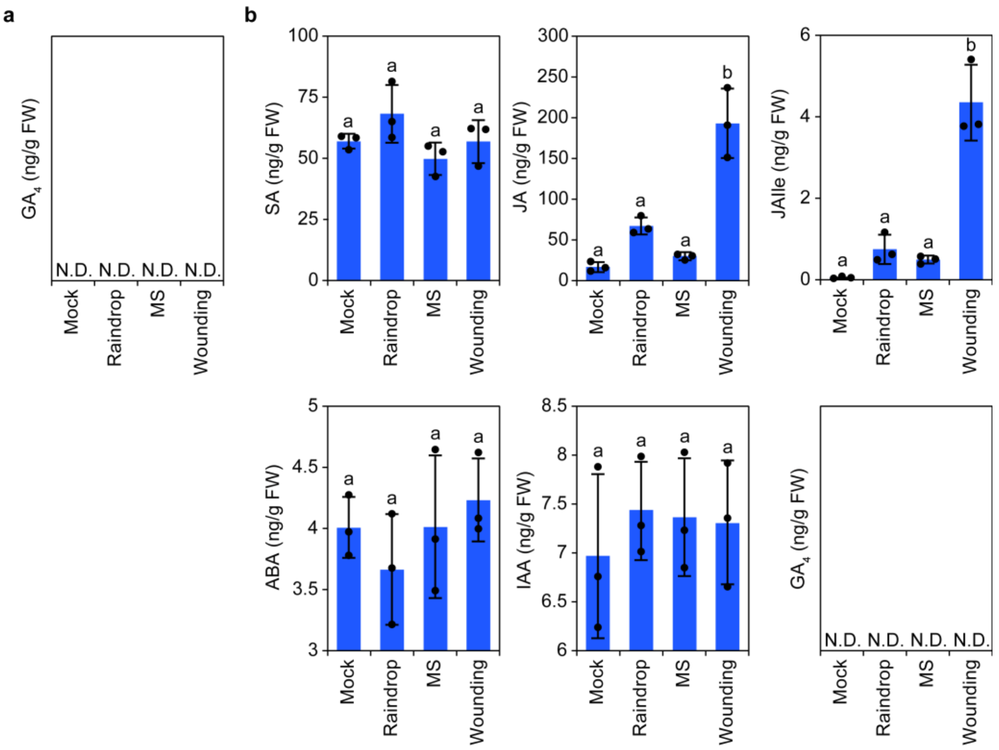

Supplementary Fig. 5 | Phytohormone measurements after raindrop or MS treatments. **a** Gibberellin A4 (GA_4) levels 5 min after treatment with 10 falling droplets (raindrop), 1 brushing (MS), and cutting (wounding). n = 6 plants examined over 3 independent experiments. Each dot indicates a biological replicate. **b** SA, JA, JA-isoleucine (JA-Ile), abscisic acid (ABA), indole-3-acetic acid (IAA), and GA_4 levels 15 min after treatment with 10 falling droplets (raindrop), 1 brushing (MS), and cutting (wounding). Data are presented as mean ± SD. Different letters above bars indicate significant differences (one-sided Tukey's multiple comparison test; $P < 0.05$). n = 5 plants examined over 3 independent experiments. Each dot indicates a biological replicate.

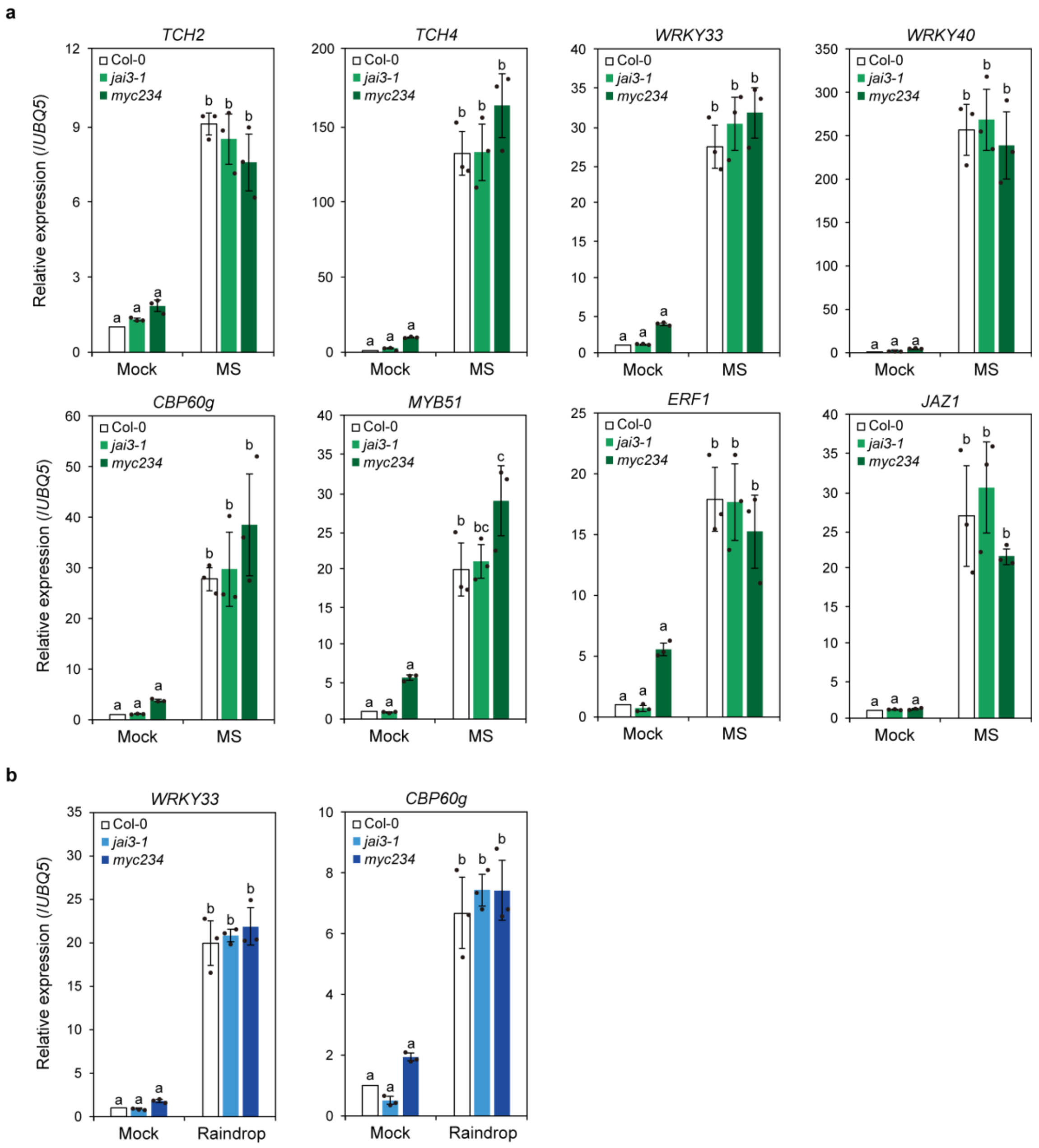

Supplementary Fig. 6 | The expression levels of some MS-induced genes are not reduced in *myc234* and *jai3-1* mutants. a,b The adaxial side of leaves was brushed once (a) or treated with 10 falling raindrops (b) and collected 15 min after treatment. Transcript levels of MS-induced and defence-related genes in 4-week-old Col-0 plants determined by RT-qPCR and normalized to *UBQ5*. Data are presented as mean ± SD. Different letters above bars indicate

significant differences (one-sided Tukey's multiple comparison test; $P < 0.05$). n = 6 plants examined over 3 independent experiments. Each dot indicates a technical replicate.

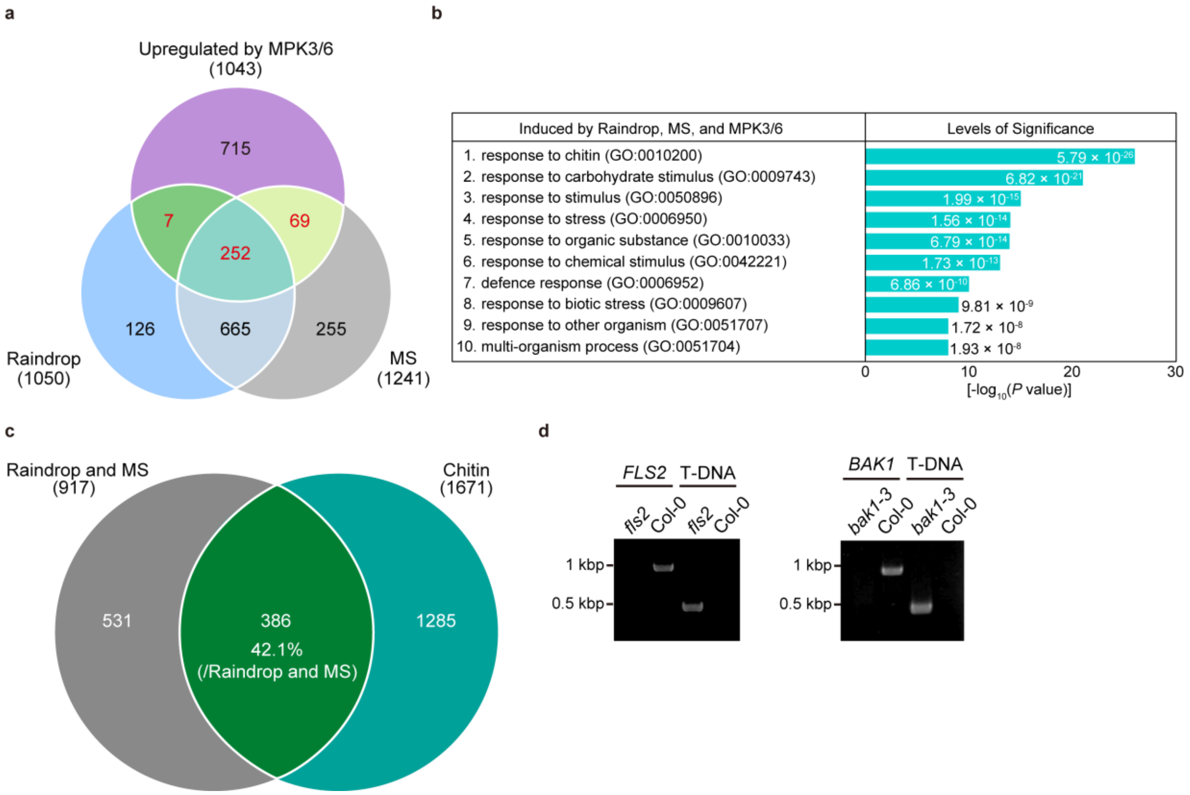

Supplementary Fig. 7 | MS-induced genes are overlapped with genes regulated by MPK3/MPK6. a Venn diagram of the overlap between genes upregulated by raindrops (1,050 genes), MS (1,241 genes), and MPK3/MPK6 (1,043 genes). A total of 328 genes (shown in red) are present in at least three groups (likelihood ratio test; $P < 0.05$). **b** Enriched GO categories of 328 genes induced by raindrops and MS through the activation of MPK3/MPK6 shown in (**a**). The top 10 categories are shown in ascending order of P values (one-sided hypergeometric test). **c** Venn diagram of the overlap between transcriptome datasets from raindrop- and MS-induced genes (917 genes) and chitin-induced genes (1,671 genes) (likelihood ratio test; $P < 0.05$). **d** Validation of the T-DNA insertion for the lines *fls2* and *bak1-3*. Genotyping PCRs were performed using the *FLS2* and *BAK1* specific primers and the forward primer of T-DNA. These PCR primers are shown in Supplementary Data 10. Similar results were obtained in 2 independent experiments using 2 biological lines.

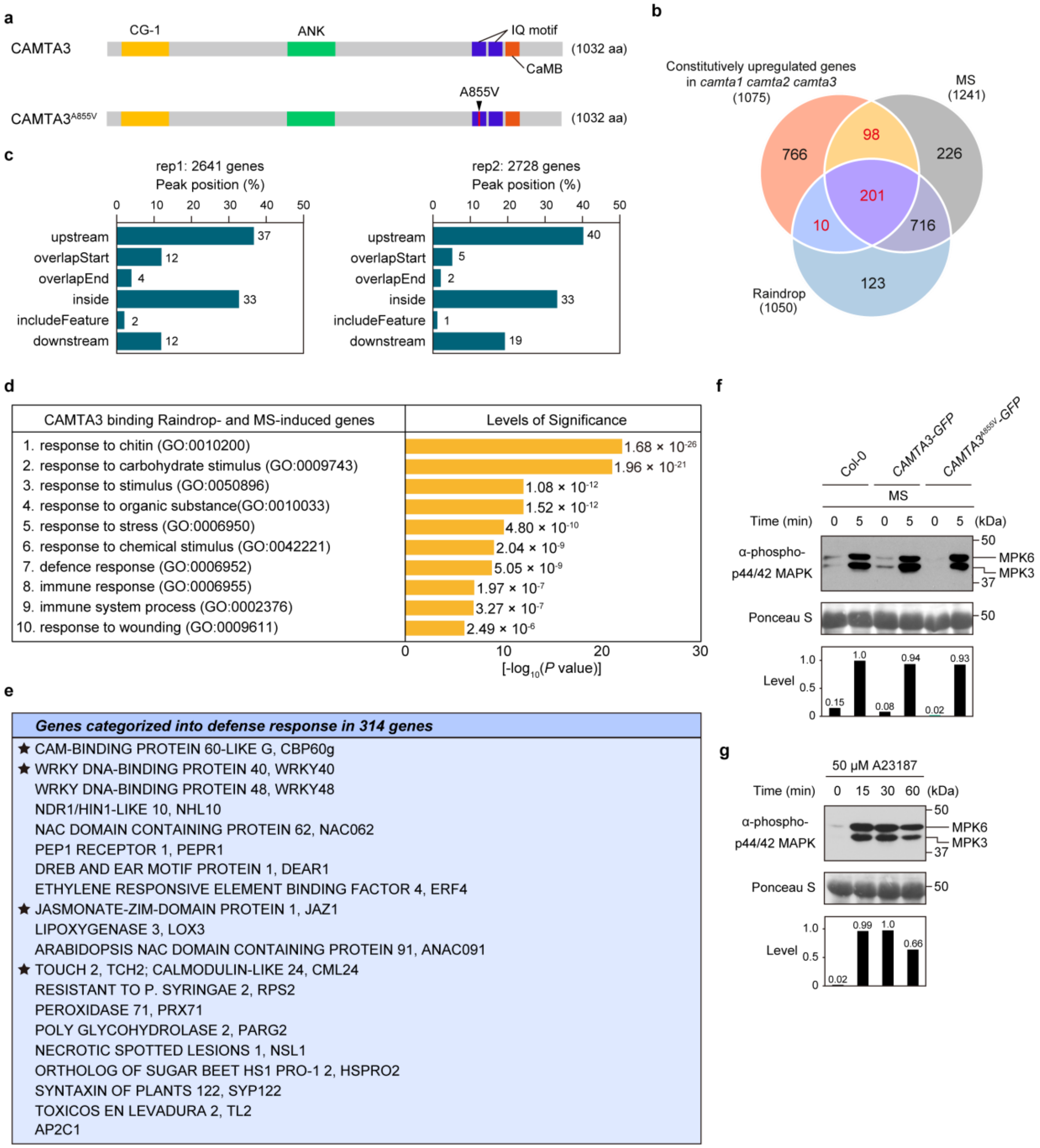
a
CAMTA3
CG-1
ANK
IQ motif
CaMB
(1032 aa)
CAMTA3A855V
A855V
(1032 aa)
b
Constitutively upregulated genes in camta1 camta2 camta3 (1075)
MS (1241)
Raindrop (1050)
766
98
226
201
10
716
123
c
rep1: 2641 genes
Peak position (%)
0 10 20 30 40 50
upstream 37
overlapStart 12
overlapEnd 4
inside 33
includeFeature 2
downstream 12
rep2: 2728 genes
Peak position (%)
0 10 20 30 40 50
upstream 40
overlapStart 5
overlapEnd 2
inside 33
includeFeature 1
downstream 19
d
CAMTA3 binding Raindrop- and MS-induced genes
Levels of Significance
1. response to chitin (GO:0010200) 1.68 × 10-26
2. response to carbohydrate stimulus (GO:0009743) 1.96 × 10-21
3. response to stimulus (GO:0050896) 1.08 × 10-12
4. response to organic substance(GO:0010033) 1.52 × 10-12
5. response to stress (GO:0006950) 4.80 × 10-10
6. response to chemical stimulus (GO:0042221) 2.04 × 10-9
7. defence response (GO:0006952) 5.05 × 10-9
8. immune response (GO:0006955) 1.97 × 10-7
9. immune system process (GO:0002376) 3.27 × 10-7
10. response to wounding (GO:0009611) 2.49 × 10-6
0 10 20 30
[-log10(P value)]
e
Genes categorized into defense response in 314 genes
★ CAM-BINDING PROTEIN 60-LIKE G, CBP60g
★ WRKY DNA-BINDING PROTEIN 40, WRKY40
WRKY DNA-BINDING PROTEIN 48, WRKY48
NDR1/HIN1-LIKE 10, NHL10
NAC DOMAIN CONTAINING PROTEIN 62, NAC062
PEP1 RECEPTOR 1, PEPR1
DREB AND EAR MOTIF PROTEIN 1, DEAR1
ETHYLENE RESPONSIVE ELEMENT BINDING FACTOR 4, ERF4
★ JASMONATE-ZIM-DOMAIN PROTEIN 1, JAZ1
LIPOXYGENASE 3, LOX3
ARABIDOPSIS NAC DOMAIN CONTAINING PROTEIN 91, ANAC091
★ TOUCH 2, TCH2; CALMODULIN-LIKE 24, CML24
RESISTANT TO P. SYRINGAE 2, RPS2
PEROXIDASE 71, PRX71
POLY GLYCOHYDROLASE 2, PARG2
NECROTIC SPOTTED LESIONS 1, NSL1
ORTHOLOG OF SUGAR BEET HS1 PRO-1 2, HSPRO2
SYNTAXIN OF PLANTS 122, SYP122
TOXICOS EN LEVADURA 2, TL2
AP2C1
f
Col-0
CAMTA3-GFP
CAMTA3A855V-GFP
MS
Time (min) 0 5 0 5 0 5 (kDa)
α-phospho-p44/42 MAPK
50
MPK6
MPK3
37
Ponceau S
50
Level
1.0
0.5
0
0.15 1.0 0.08 0.94 0.02 0.93
g
50 μM A23187
Time (min) 0 15 30 60 (kDa)
α-phospho-p44/42 MAPK
50
MPK6
MPK3
37
Ponceau S
50
Level
1.0
0.5
0
0.02 0.99 1.0 0.66

Supplementary Fig. 8 | MS-induced genes regulated by CAMTAs show enrichment in categories associated with plant immunity. a Diagram of CAMTA3 and CAMTA3^{A855V} proteins. CG-1, DNA (CGCG box)-binding domain; ANK, ankyrin repeat domain; IQ and CaMB, calmodulin (CaM)-binding domain. **b** Venn diagram of transcriptome datasets obtained by brushing (1,241 genes) and raindrops (1,050 genes) and constitutively upregulated genes in the *camta1 camta2 camta3* triple mutant (1,075 genes) (likelihood ratio test; P < 0.05). Overlap with MS-induced genes; constitutively upregulated genes in the *camta1 camta2 camta3* triple mutant (28.7%; 309/1,075 genes). The 201 shared upregulated genes include *WRKY33*, *WRKY40*, and *CBP60g*. **c** Positions of the 2,641 and 2,728 CAMTA3-binding peaks in 2,011 genes relative to the annotated nearest transcription start site (TSS). Upstream, peak resides upstream of the feature; overlapStart, peak overlaps with the start of the feature; overlapEnd, peak overlaps with the end of the feature; inside, peak resides inside the feature; includeFeature, peak includes the feature entirely; downstream, peak resides downstream of the feature. **d** Enriched Gene Ontology categories of 314 CAMTA3 target genes shown in Fig. 3d. The top 10 categories are shown in ascending order of P values (one-sided hypergeometric test). **e** Representative MS-induced defence genes in 314 CAMTA target genes. Black stars indicate the RT-qPCR marker genes used in this work. **f** Brush treatment (MS) induces MAPK activation in Col-0, *camta2 camta3 CAMTA3-GFP*, and *camta2 camta3 CAMTA3^{A855V}-GFP*. Total proteins were extracted from 4-week-old plants 5 min after MS treatment (4 brushing) and detected by immunoblot analysis with anti-phospho-p44/42 MAPK antibodies. Relative phosphorylation levels are shown below each blot. Similar results were obtained in 3 independent experiments. **g** Cytosolic Ca^{2+}-induced MAPK activation in Col-0. Total proteins were extracted from 12-day-old seedlings treated with 50 µM calcium ionophore A23187 and detected by immunoblot analysis with anti-p44/42 MAPK antibodies. Relative phosphorylation levels are shown below each blot. Similar results were obtained in 6 independent experiments.

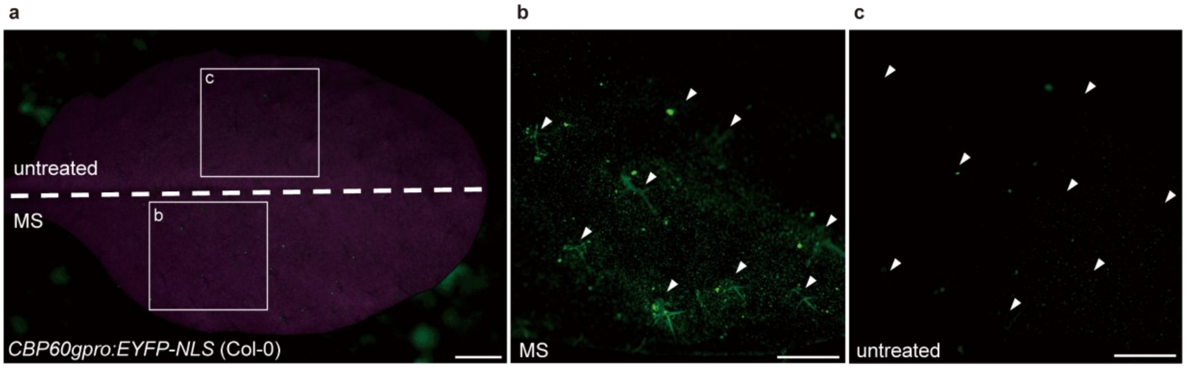

Supplementary Fig. 9 | MS induces the expression of *CBP60g* in cells surrounding trichomes. a-c YFP fluorescence from the whole leaf of *CBP60gpro:EYFP-NLS* (Col-0) with (MS; bottom half) or without brushing (untreated; top half) (**a**), with zoomed-in views of MS (**b**) and untreated (**c**) areas. Arrowheads indicate trichomes (**b**, **c**). Scale bars, 0.5 mm (**a**), 0.3 mm (**b**, **c**). Similar results were obtained in 2 independent experiments.

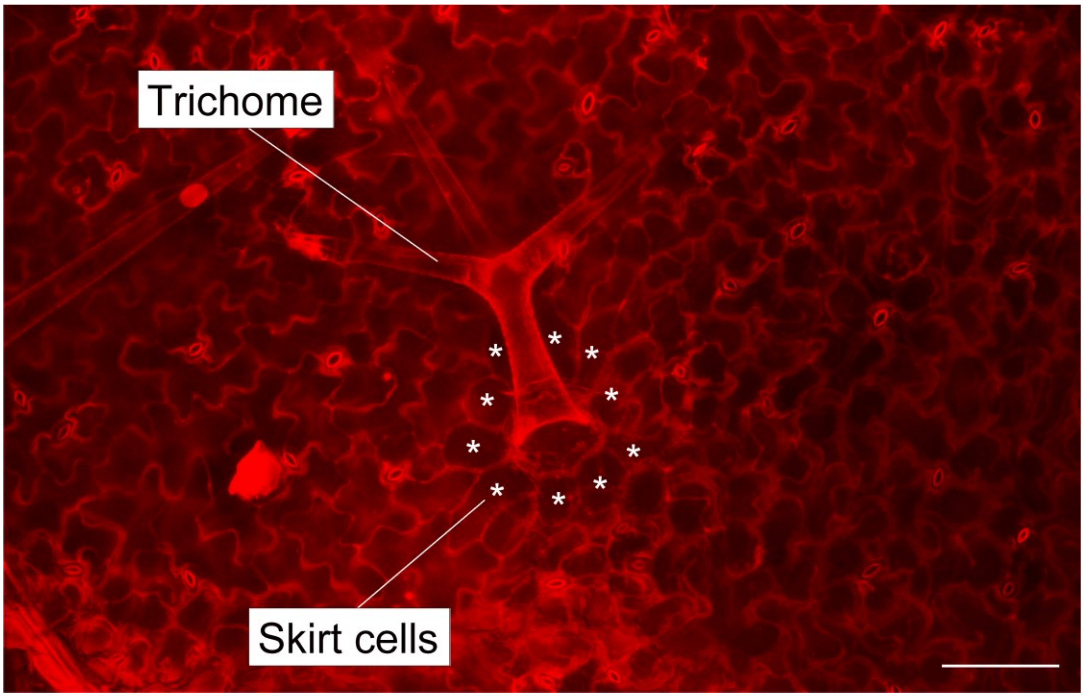

Supplementary Fig. 10 | Trichomes are surrounded by the skirt cells. Confocal imaging showing propidium iodide staining of the leaf cell wall. Asterisks indicate skirt cells. Scale bars, 0.1 mm. Similar results were obtained in 3 independent experiments.

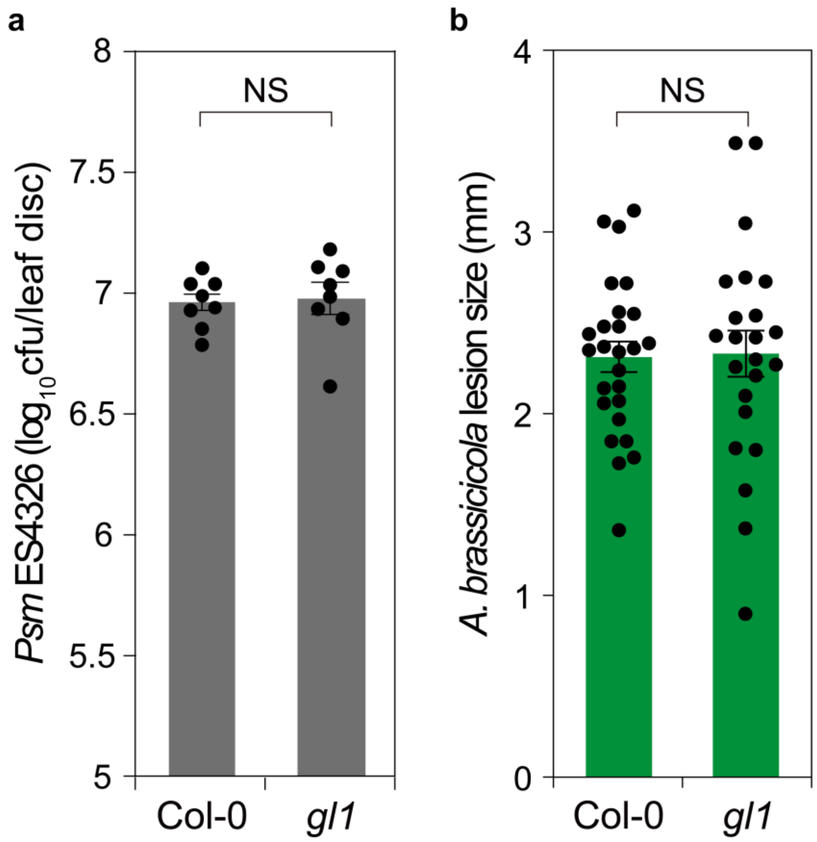

Supplementary Fig. 11 | The local resistance against *Psm* ES4326 and *A. brassicicola* Ryo-1 is intact in the *gl1* mutant. a Growth of *Psm* ES4326 in Col-0 and *gl1* leaves 2 days after inoculation. Error bars represent SE. NS, not significant. Cfu, colony-forming units. n = 8 samples examined over 3 independent experiments. Each dot indicates a biological replicate. **b** Disease progression of *A. brassicicola* in Col-0 and *gl1* leaves 3 days after inoculation. Error bars represent SE. NS, not significant. Col-0: n = 26, *gl1*: n = 23 samples examined over 3 independent experiments. Each dot indicates a biological replicate.

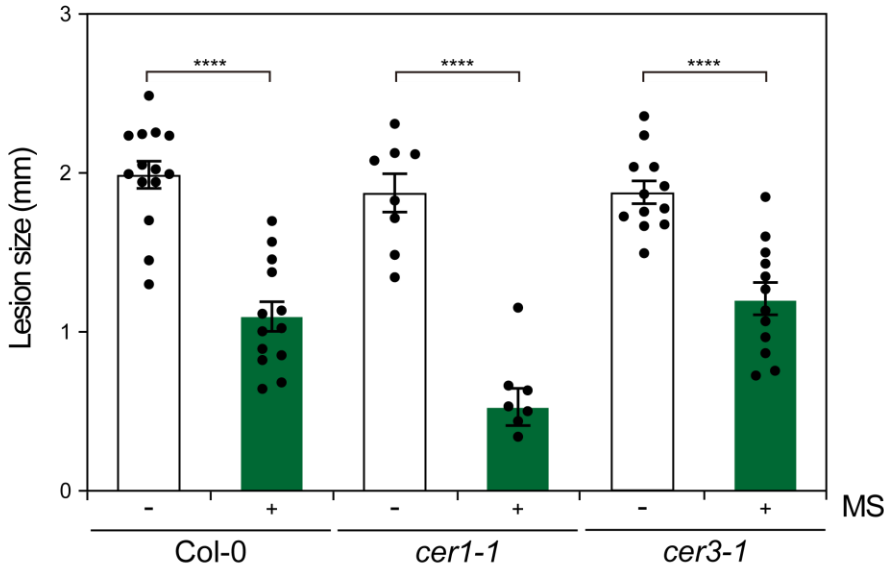

Supplementary Fig. 12 | Immune response against *A. brassicicola* Ryo-1 is enhanced by MS in cuticle- and SAR-defective mutants *cer1* and *cer3*. Disease progression of *A. brassicicola* in Col-0, *cer1-1*, and *cer3-1* leaves 3 days after inoculation with (+) or without (–) MS (4 brushing) pretreatment. Error bars represent SE. Asterisks indicate significant difference (one-sided Šídák's multiple comparison test; ****$P < 0.0001$). Col-0 (–): n = 14, Col-0 (+): n = 13, *cer1-1* (–): n = 8, *cer1-1* (+): n = 7, *cer3-1* (–): n = 12, *cer3-1* (+): n = 12 samples examined over 3 independent experiments. Each dot indicates a biological replicate.

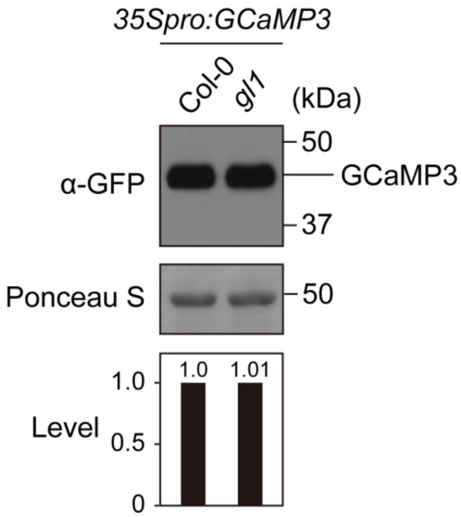

Supplementary Fig. 13 | The expression levels of GCaMP3 protein in *35Spro*:*GCaMP3*/Col-0 and *35Spro*:*GCaMP3*/*gl1*. Total proteins were extracted from 4-week-old plants and detected by immunoblot analysis with anti-GFP antibodies. Relative protein levels are shown below each blot. Similar results were obtained in 4 independent experiments.

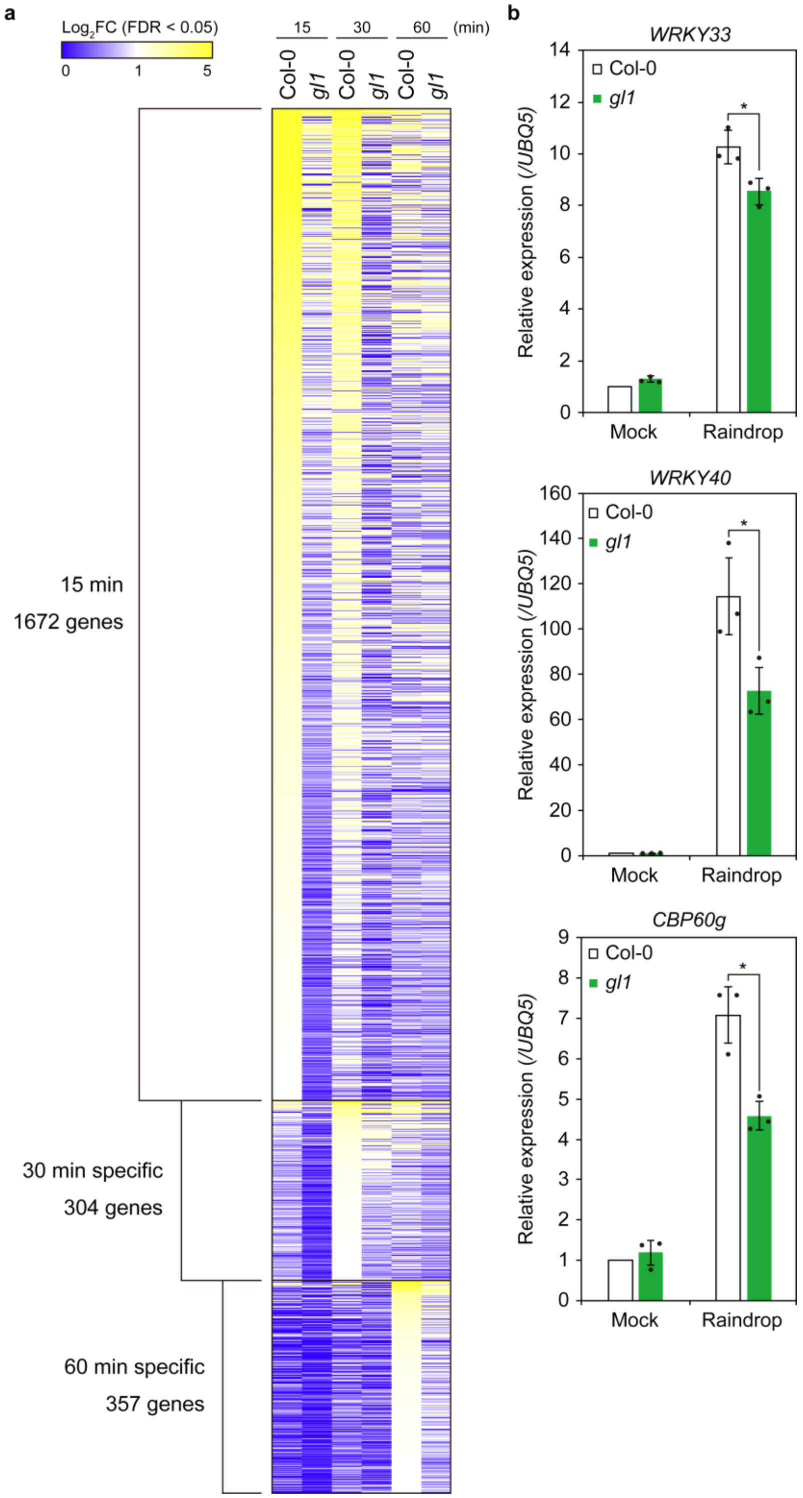

a
Log2FC (FDR < 0.05)
0
1
5
15
30
60
(min)
Col-0
gl1
Col-0
gl1
Col-0
gl1
15 min
1672 genes
30 min specific
304 genes
60 min specific
357 genes
b
WRKY33
WRKY40
CBP60g
Col-0
gl1
Relative expression (/UBQ5)
Mock
Raindrop
*

Supplementary Fig. 14 | Trichomes amplify the expression of MS-induced genes. a Heatmap of differentially expressed genes obtained from transcriptome datasets for Col-0 and *gl1* plants treated by MS (4 brushing). Genes upregulated 15 min after brushing: 1,672 genes; unique induced 30 min after brushing: 304 genes; unique induced 60 min after brushing: 357 genes. See also Fig. 5c, d. **b** Four-week-old Col-0 and *gl1* plants were treated with 1 falling droplet. Transcript levels of *WRKY33*, *WRKY40*, and *CBP60g* 15 min after treatment with 1 falling droplet were determined using RT-qPCR and normalized to *UBQ5*. Data are presented as mean ± SD. Asterisks indicate significant difference (one-sided Tukey's multiple comparison test; *$P < 0.05$). n = 6 plants examined over 3 independent experiments. Each dot indicates a technical replicate.

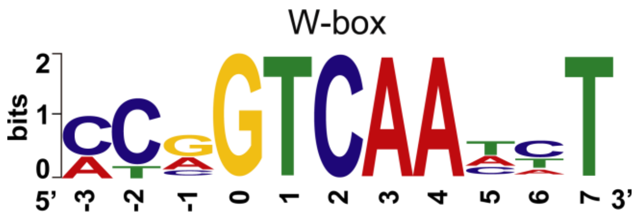

Supplementary Fig. 15 | Promoter analysis of MAPK-regulated 252 genes among 917 raindrop- and MS-induced genes. The WRKY-binding W-box (TTGACC) was overrepresented among these genes.

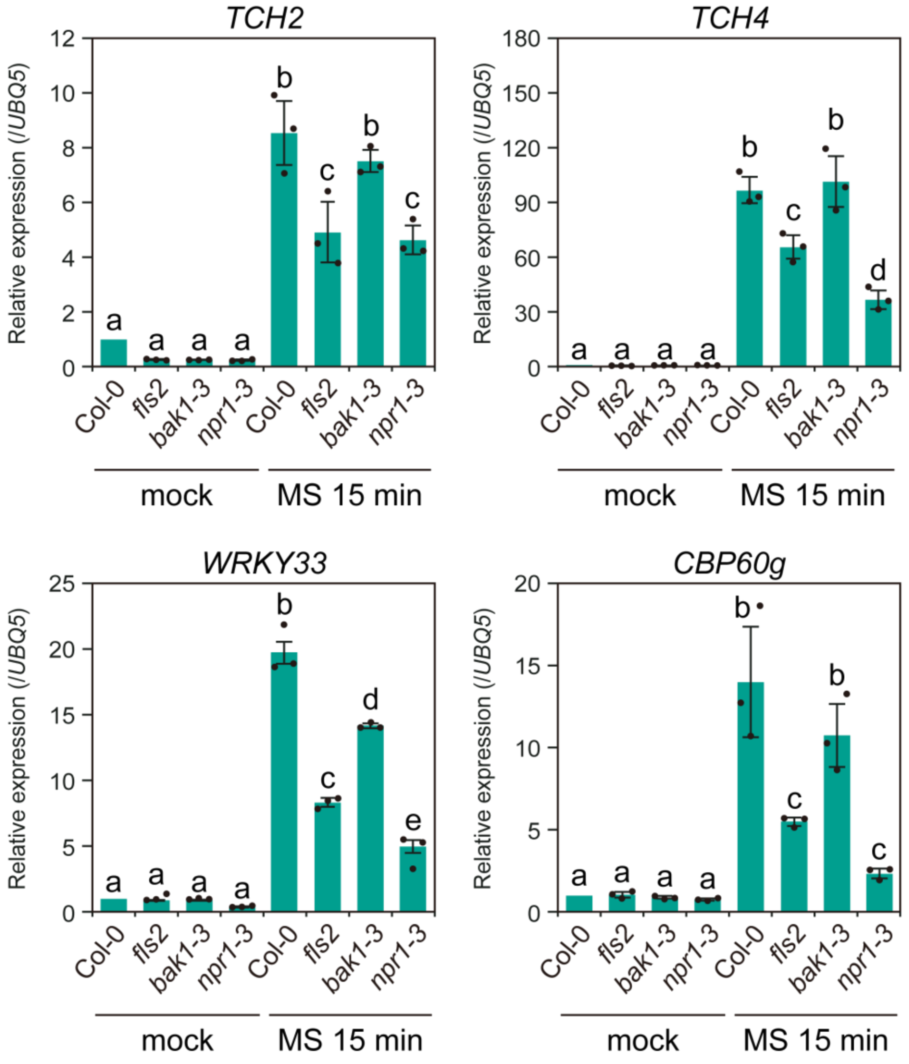

Supplementary Fig. 16 | MS-induced gene expression in various defence mutants. Four-week-old Col-0, *fls2*, *bak1-3*, and *npr1-3* plants were brushed 4 times. Transcript levels of *TCH2*, *TCH4*, *WRKY33*, and *CBP60g* 15 min after brushing were determined using RT-qPCR and normalized to *UBQ5*. Data are presented as mean ± SD. Different letters above bars indicate significant differences (one-sided Tukey's multiple comparison test; $P < 0.05$). n = 6 plants examined over 3 independent experiments. Each dot indicates a technical replicate.
