# Supplementary material for: Mechanosensory trichome cells evoke a mechanical stimuli–induced immune response in Arabidopsis thaliana
Source: Nat Commun. 2022 Mar 8;13:1216. doi: 10.1038/s41467-022-28813-8 (PMC8904797; doi:10.1038/s41467-022-28813-8)
Supplement: Supplementary file 3 — Description of Additional Supplementary Files [file 41467_2022_28813_MOESM3_ESM.pdf]

Description of Additional Supplementary Files

File name: Supplementary Data 1

Description: The list of 1,050 genes upregulated 15 minutes after treatment of Col-0 leaves with 10 falling raindrops. This list contains locus identifier, $\log_2$FC, *P* value, and gene description ($\log_2$FC ≥ 1, likelihood ratio test; $P < 0.05$)

File name: Supplementary Data 2

Description: Gene Ontology terms of 1,050 genes listed in Supplementary Data 1. This list contains GO term description and *P* value generated by BiNGO.

File name: Supplementary Data 3

Description: The list of 1,241 genes upregulated 15 minutes after treatment of Col-0 leaves with MS (one brushing). This list contains locus identifier, $\log_2$FC, *P* value, and gene description ($\log_2$FC ≥ 1, likelihood ratio test; $P < 0.05$).

File name: Supplementary Data 4

Description: Gene Ontology terms of 1,241 genes listed in Supplementary Data 3. This list contains GO term description and *P* value generated by BiNGO.

File name: Supplementary Data 5

Description: The list of 328 genes upregulated by 10 falling raindrops, MS (one brushing), and MPK3/MPK6. This list contains locus identifier and gene description ($\log_2$FC ≥ 1, likelihood ratio test; $P < 0.05$).

File name: Supplementary Data 6

Description: Gene Ontology terms of 328 genes listed in Supplementary Data 5. This list contains GO term description and *P* value generated by BiNGO.

File name: Supplementary Data 7

Description: The list of overlapped genes among constitutively upregulated genes in the *camta1 camta2 camta3* mutant, 10 falling raindrops- and MS (one brushing)-induced genes in Col-0. This list contains locus identifier and gene description ($\log_2$FC ≥ 1, likelihood ratio test; $P < 0.05$).

File name: Supplementary Data 8

The list of CAMTA3(A855V)-binding peaks/genes in ChIP-seq analysis.

File name: Supplementary Data 9

Description: The list of genes upregulated 15, 30, and 60 minutes after brushing 4 times in Col-0 and *gl1* mutant. This list contains locus identifier, $\log_2$FC, *P* value, and gene description ($\log_2$FC ≥ 1, likelihood ratio test; $P < 0.05$).

File name: Supplementary Data 10

Description: The list includes the primer names and sequences used for RT-qPCR, genotyping PCR, and sequencing.

File name: Supplementary Data 11

Description: The list includes the information of transcriptome datasets for meta-analysis.

File name: Supplementary Movie 1

Description: Ca^{2+} imaging using *35Spro:GCaMP3* (Col-0). Adaxial side of the leaf surface was gently brushed. Intercellular calcium waves propagated concentrically from the trichomes. Scale bar, 1.0 mm.

File name: Supplementary Movie 2

Description: Ca^{2+} imaging using *35Spro:GCaMP3* (Col-0). Trichome necks were manually flicked by a silver chloride wire. Intercellular calcium waves propagated concentrically from the trichomes. Scale bar, 0.2 mm.

File name: Supplementary Movie 3

Description: Ca^{2+} imaging using *35Spro:GCaMP3* (Col-0). Side views of the trichome that were manually flicked by a silver chloride wire. The base of trichomes showed a rapid and transient increase in $[Ca^{2+}]_{cyt}$ before the concentric propagation of calcium waves. Scale bar, 0.1 mm.

File name: Supplementary Movie 4

Description: Ca^{2+} imaging using *35Spro:GCaMP3* (Col-0). Adaxial side of the whole leaf was gently brushed. Intercellular calcium waves were obviously detected. Scale bar, 0.5 mm.

File name: Supplementary Movie 5

Description: Ca^{2+} imaging using *35Spro:GCaMP3 (gl1)*. Adaxial side of the whole leaf was gently brushed. Intercellular calcium waves were not clearly detected. Scale bar, 0.5 mm.
