# Supplementary material for: Mechanosensory trichome cells evoke a mechanical stimuli–induced immune response in Arabidopsis thaliana
Source: Nat Commun. 2022 Mar 8;13:1216. doi: 10.1038/s41467-022-28813-8 (PMC8904797; doi:10.1038/s41467-022-28813-8)
Supplement: Supplementary file 20 — Reporting Summary [file 41467_2022_28813_MOESM20_ESM.pdf]

nature portfolio

Corresponding author(s): Yasuomi Tada and Mika Nomoto

Last updated by author(s): Jan 25, 2022

Reporting Summary

Nature Portfolio wishes to improve the reproducibility of the work that we publish. This form provides structure for consistency and transparency in reporting. For further information on Nature Portfolio policies, see our Editorial Policies and the Editorial Policy Checklist.

Statistics

For all statistical analyses, confirm that the following items are present in the figure legend, table legend, main text, or Methods section.

n/a	Confirmed	
☐	☒	The exact sample size (*n*) for each experimental group/condition, given as a discrete number and unit of measurement
☐	☒	A statement on whether measurements were taken from distinct samples or whether the same sample was measured repeatedly
☐	☒	The statistical test(s) used AND whether they are one- or two-sided<br>*Only common tests should be described solely by name; describe more complex techniques in the Methods section.*
☒	☐	A description of all covariates tested
☐	☒	A description of any assumptions or corrections, such as tests of normality and adjustment for multiple comparisons
☐	☒	A full description of the statistical parameters including central tendency (e.g. means) or other basic estimates (e.g. regression coefficient) AND variation (e.g. standard deviation) or associated estimates of uncertainty (e.g. confidence intervals)
☐	☒	For null hypothesis testing, the test statistic (e.g. *F*, *t*, *r*) with confidence intervals, effect sizes, degrees of freedom and *P* value noted<br>*Give P values as exact values whenever suitable.*
☒	☐	For Bayesian analysis, information on the choice of priors and Markov chain Monte Carlo settings
☒	☐	For hierarchical and complex designs, identification of the appropriate level for tests and full reporting of outcomes
☐	☒	Estimates of effect sizes (e.g. Cohen's *d*, Pearson's *r*), indicating how they were calculated

Our web collection on statistics for biologists contains articles on many of the points above.

Software and code

Policy information about availability of computer code

Data collection	RNA-seq: BaseSpace (Illumina), EdgeR package on the web Degust<br>Go analysis: BiNGO (Cytoscape)<br>ChIP-seq: Bowtie2, SAMtools, IGV, MACS2, BEDtools<br>Cis analysis: MEME-ChIP<br>LC-MS analysis: OpenLab CDS 2 (Agilent Technologies Inc.)
Data analysis	GraphPad Prism 9 was used in this study.

For manuscripts utilizing custom algorithms or software that are central to the research but not yet described in published literature, software must be made available to editors and reviewers. We strongly encourage code deposition in a community repository (e.g. GitHub). See the Nature Portfolio guidelines for submitting code & software for further information.

Data

Policy information about availability of data

All manuscripts must include a data availability statement. This statement should provide the following information, where applicable:

- Accession codes, unique identifiers, or web links for publicly available datasets
- A description of any restrictions on data availability
- For clinical datasets or third party data, please ensure that the statement adheres to our policy

The authors declare that all data supporting the findings of this study are available within this article and its Supplementary Information files. RNA-seq and ChIP-seq data have been deposited in the DDBJ Sequence Read Archive at the DNA Data Bank (http://www.ddbj.nig.ac.jp/) with the accession numbers DRA011970,

DRA009248 and DRA011123.
All other data are available from the corresponding authors upon request.

Field-specific reporting

Please select the one below that is the best fit for your research. If you are not sure, read the appropriate sections before making your selection.

☒ Life sciences ☐ Behavioural & social sciences ☐ Ecological, evolutionary & environmental sciences

For a reference copy of the document with all sections, see nature.com/documents/nr-reporting-summary-flat.pdf

Life sciences study design

All studies must disclose on these points even when the disclosure is negative.

Sample size	Sample sizes were determined based on previous reports and best practices.
Data exclusions	No data points were excluded.
Replication	Experimental findings were reliably reproduced.
Randomization	Due to the nature of the experimental setup blinding/randomization was not practical.
Blinding	Due to the nature of the experimental setup blinding/randomization was not practical.

Reporting for specific materials, systems and methods

We require information from authors about some types of materials, experimental systems and methods used in many studies. Here, indicate whether each material, system or method listed is relevant to your study. If you are not sure if a list item applies to your research, read the appropriate section before selecting a response.

Materials & experimental systems

n/a	Involved in the study
☐	☒ Antibodies
☒	☐ Eukaryotic cell lines
☒	☐ Palaeontology and archaeology
☒	☐ Animals and other organisms
☒	☐ Human research participants
☒	☐ Clinical data
☒	☐ Dual use research of concern

Methods

n/a	Involved in the study
☐	☒ ChIP-seq
☒	☐ Flow cytometry
☒	☐ MRI-based neuroimaging

Antibodies

Antibodies used	Rabbit polyclonal anti-GFP (ChIP grade), Abcam, Cat# ab290, 1:600 dilution for ChIP, 1:4000 dilution for immunoblot<br>Rabbit IgG polyclonal-Isotype control, Abcam, Cat# ab37415, 1:600 dilution for ChIP<br>Anti-phospho-p44/42 MAPK polyclonal antibody, Cell Signaling Technology, Cat# 9101, 1:1000 dilution for immunoblot<br>Goat anti-rabbit IgG(H+L)-HRP conjugate, BIO-RAD, Cat# 170-6515, 1:2000 dilution for immunoblot
Validation	Rabbit polyclonal anti-GFP (ChIP grade): Validation info is described on the supplier's web page, https://www.abcam.co.jp/gfp-antibody-ab290.html.<br>Anti-phospho-p44/42 MAPK polyclonal antibody: Validation info is available in the cited paper ""Galletti et al., Plant Physiol., 2011, 157, 804-814.

ChIP-seq

Data deposition

☒ Confirm that both raw and final processed data have been deposited in a public database such as GEO.

☐ Confirm that you have deposited or provided access to graph files (e.g. BED files) for the called peaks.

Data access links<br>*May remain private before publication.*	ChIP-seq data have been deposited in the DDBJ Sequence Read Archive at the DNA Data Bank (http://www.ddbj.nig.ac.jp/) with the accession numbers DRA011123. Direct Link: https://ddbj.nig.ac.jp/public/ddbj_database/dra/fastq/DRA011/DRA011123/

Files in database submission: DRR256173.fastq.bz2, DRR256174.fastq.bz2, DRR256175.fastq.bz2

Genome browser session (e.g. UCSC): DDBJ Sequence Read Archive at the DNA Data Bank (http://www.ddbj.nig.ac.jp/)

Methodology

Replicates: CAMTA3AV_Input, CAMTA3AV_IP rep1, CAMTA3AV_IP rep2

Sequencing depth:

The sequencing informarion of CAMTA3AV_Input, CAMTA3AV_IP rep1, and CAMTA3AV_IP rep2 were described below.
<CAMTA3AV_Input>
21228575 reads; of these:
 21228575 (100.00%) were unpaired; of these:
  10583450 (49.85%) aligned 0 times
  7354589 (34.64%) aligned exactly 1 time
  3290536 (15.50%) aligned >1 times
50.15% overall alignment rate

<CAMTA3AV_IP rep1>
22636185 reads; of these:
 22636185 (100.00%) were unpaired; of these:
  2791360 (12.33%) aligned 0 times
  14254932 (62.97%) aligned exactly 1 time
  5589893 (24.69%) aligned >1 times
87.67% overall alignment rate

<CAMTA3AV_IP rep2>
29756309 reads; of these:
 29756309 (100.00%) were unpaired; of these:
  4000788 (13.45%) aligned 0 times
  16798682 (56.45%) aligned exactly 1 time
  8956839 (30.10%) aligned >1 times
86.55% overall alignment rate

All samples were sequenced single-end 81 bp.

Antibodies: Rabbit polyclonal anti-GFP (Abcam, Cat# ab290); Rabbit IgG, polyclonal-Isotype Control (Abcam, Cat# ab37415)

Peak calling parameters:

$ macs2 callpeak -c CAMTA3AV_Input.sort.bam -t CAMTA3AV_IP rep1.sort.bam -n CAMTA3AV_IP rep1 -p 0.05 -g 1.19e8
$ macs2 callpeak -c CAMTA3AV_Input.sort.bam -t CAMTA3AV_IP rep2.sort.bam -n CAMTA3AV_IP rep2 -p 0.05 -g 1.19e8

Data quality: MACS2 Score > 30

Software: Bowtie2, SAMtools, IGV, deepTools, MACS2, R package Biocondctor, R package ChIPpeakAnno, Bedtools, MEME-ChIP
